# Advances and Challenges in the Integration of Digital Technologies in Complete Dentures: A Narrative Literature Review

**DOI:** 10.4317/jced.62598

**Published:** 2025-05-01

**Authors:** Jéssica de Oliveira Alvarenga Freire, Adriana Cristina Zavanelli, José Vitor Quinelli Mazaro, Ricardo Alexandre Zavanelli, Rodrigo Sversut de Alexandre, Otavio Marino dos Santos Neto

**Affiliations:** 1 São Paulo State University (UNESP) - School of Dentistry, Araçatuba - Rua José Bonifácio, 1193 - Vila Mendonça - Araçatuba - 16015-050 – Brazil; 2 Federal University of Goiás (UFG), Faculty of Dentistry - Av. Universitária, s/n - Setor Leste Universitário - Goiânia - 74605-020 - Brazil; 3 Federal University of Santa Catarina (UFSC), Department of Dentistry - Rua Delfino Conti, s/n - Trindade - Florianópolis - 88040-900 - Brazil

## Abstract

**Background:**

The fully edentulous population in Brazil comprises approximately 22 million individuals, highlighting the importance of their functional, aesthetic, and psychosocial rehabilitation. Complete dentures (CD) remain the most accessible treatment option for these patients, supported by well-established scientific techniques. The introduction of the digital workflow in Dental Prosthetics has streamlined production, reducing clinical time and material consumption. While the digital workflow is well-established in fixed prosthetics, its application in CD still lacks equivalent validation. Given the challenges of achieving retention, stability, comfort, and aesthetics, it remains uncertain whether the digital workflow can deliver outcomes comparable to those of the conventional analog method.

**Material and Methods:**

A qualitative literature review was conducted through searches in the PUBMED database using the terms ‘digital complete denture,’ ‘complete denture,’ ‘scanning,’ ‘intraoral scanning,’ and ‘CAD-CAM,’ covering the period from 1994 to 2024. Initially, articles were screened based on their titles. Subsequently, the abstracts of the selected studies were analyzed, and those deemed relevant were read in full.

**Results:**

Intraoral scanning of edentulous ridges exhibits accuracy comparable to conventional impressions in static áreas, however, reproducing dynamic regions remains a challenge. The adaptation of digital complete dentures (DCDs) obtained through intraoral scanning is considered clinically acceptable, although retention outcomes remain controversial. Milled polymethyl methacrylate (PMMA) demonstrates mechanical properties equal to or superior to those of thermopolymerizable PMMA. In contrast, 3D-printed resins exhibit mechanical properties and longevity similar to or inferior to both milled and thermopolymerizable PMMA. Overall, patient acceptance of DCDs appears to be positive.

**Conclusions:**

DCDs can be manufactured more efficiently and rapidly, with clinical outcomes and patient acceptance comparable to conventional CDs. However, further scientific evidence and long-term validation are still required.

** Key words:**Complete dentures, CAD/CAM, digital complete dentures, digital workflow, intraoral scanning.

## Introduction

Edentulism compromises essential functions such as mastication and phonetics, while also impacting aesthetics, social interaction, and psychological well-being, making prosthetic rehabilitation fundamental. Treatment modalities include mucosa-supported complete dentures, implant-retained overdentures, and implant-supported fixed prostheses (Brånemark protocol). Fixed prostheses, such as the Brånemark protocol, offer better masticatory performance and comfort but remain costly and less accessible, especially for low-income populations. In 2021, approximately 353 million people worldwide were completely edentulous, highlighting the significant demand for complete dentures (1). Given this scenario, it is essential for prosthodontists to deliver high-quality treatments, ensuring effective and reliable rehabilitations.

The foundational concepts of modern complete dentures were established in the 18th century by *Pi*erre Fauchard, the father of modern dentistry. Many materials and techniques used today were developed long ago, resulting in well-established, scientifically grounded protocols for complete denture fabrication (2). However, crafting a satisfactory complete denture remains challenging. Numerous factors influence the final outcome and success of the prosthesis, including those related to the dentist, the dental laboratory, and the patient. The traditional process of complete denture fabrication involves five main steps: anatomical impression, functional impression, wax rim orientation and intermaxillary record, aesthetic-functional trial, and delivery.

CAD/CAM (Computer-Aided Design/Computer-Aided Manufacturing) technology uses specialized software for 3D design and manufacturing, which can be carried out through 3D printing (additive method) or milling (subtractive method) (3). In dentistry, CAD/CAM was initially developed for fixed prostheses. In September 1985, at the University of Zurich, the first chairside CAD/CAM feldspathic ceramic inlay was fabricated and adhesively cemented (4). Since then, advances in scanning and milling processes have improved the accuracy and efficiency of the digital workflow, resulting in minimal discrepancies compared to analog methods and establishing CAD/CAM as a reliable option for fabricating fixed prostheses (5).

However, scanning edentulous arches still presents challenges, particularly in accurately capturing the viscoelasticity of soft tissues, which can compromise scan accuracy and, consequently, the final outcome of complete dentures (6,7). As an alternative to intraoral scanning, plaster models or impressions can be digitized. Complete dentures can be fabricated using 3D printers, employing specific resins, or milling machines, using pre-manufactured polymethyl methacrylate (PMMA) blocks (3).

Despite advancements, questions remain regarding the efficacy of fabricating complete dentures through the digital workflow. Recent studies have sought to overcome these limitations and refine the techniques used. In this context, the aim of this study is to compare the efficacy of complete dentures fabricated through digital workflows with those produced using analog methods, considering aspects such as accuracy, functionality, and clinical predictability through a narrative literature review.

## Material and Methods

A comprehensive qualitative literature review was conducted, involving a search for relevant articles in the PubMed/Medline, Scopus, and Embase databases. The search utilized the following terms: “digital complete denture,” “scanning,” “intraoral scanning,” and “CAD-CAM,” combined using the Boolean operators “OR” and “AND.” The period considered for study selection ranged from 1994 to 2024.

During the article selection process, studies were initially screened by carefully reading the titles to identify those most relevant to the review’s objective. Following this initial screening, the abstracts of the selected articles were analyzed in detail. Studies whose abstracts indicated relevance to the review were then read in full for a more thorough evaluation to ensure they met the defined inclusion criteria. Articles reporting the history and/or description of techniques and/or comparisons between conventional and digital dentures regarding various properties were selected. Systematic reviews were prioritized for inclusion.

## Results

Common Concepts of Complete Dentures in Both Analog and Digital Workflows

Complete dentures are based on well-established principles, which also underpin digital complete dentures. A strong understanding of maxillary and mandibular anatomy is crucial for effective impression-making and, consequently, the fabrication of functional complete dentures. The “bearing area,” which is the region in contact with the denture base, includes structures such as the labial frenum, vestibular sulcus, hamular notch, and tuberosity in the maxillary arch, as well as the retromolar pad, external oblique ridge, and retromolar fossa in the mandibular arch (8). These areas are fundamental for ensuring denture stability, retention, and comfort (8). Additionally, knowledge of the characteristics of different regions of an edentulous ridge is essential.

**Figure 1:**
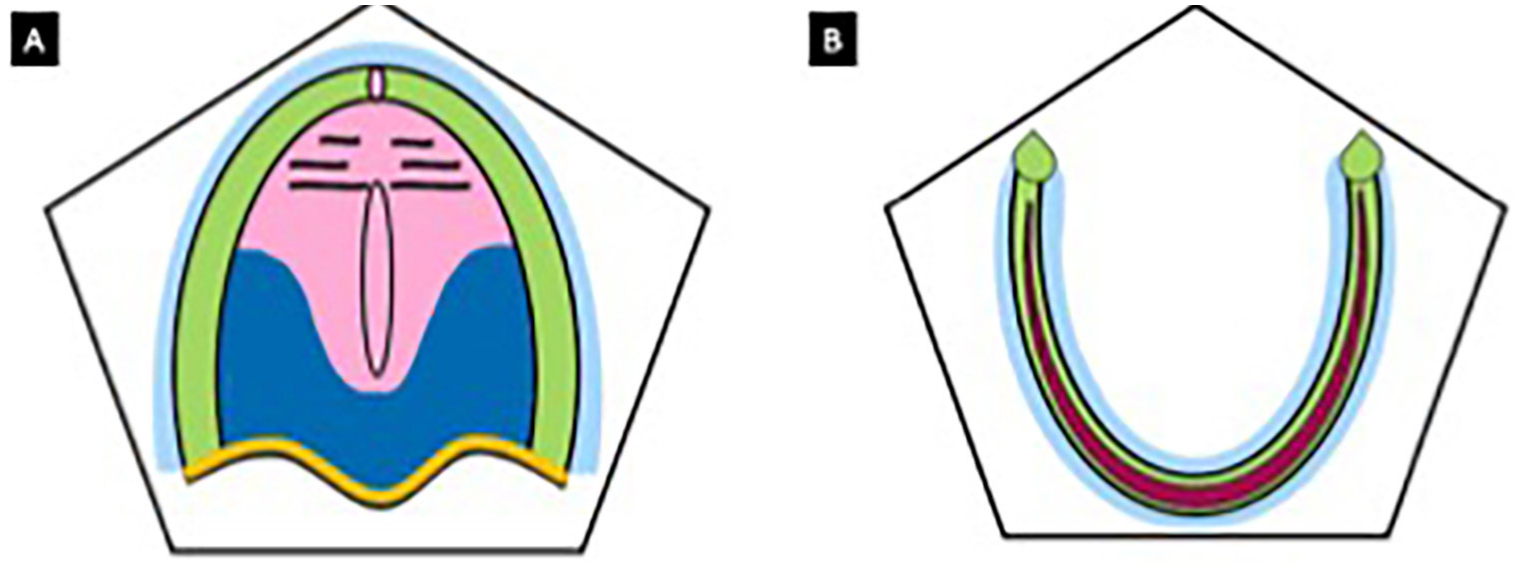
Pendleton Zones. A. Maxilla: primary support zone (green), secondary support zone (dark blue), peripheral seal zone (light blue), posterior locking zone (yellow), relief zone (pink); B. Mandible: primary support zone (dark pink), secondary support zone (green), peripheral seal zone (blue).

Pendleton described five support zones for complete dentures (9):

• Primary Support Zone: Corresponding to the crest of the alveolar ridge, it is the primary area responsible for denture support and bears the majority of masticatory forces.

• Secondary Support Zone: Located on the slopes of the ridge, it plays a key role in immobilizing the denture horizontally.

• Peripheral Seal Zone: Around the edges of the vestibule, it ensures a seal that prevents air entry and aids retention.

• Posterior Seal Zone: A thickened area in the soft palate that enhances retention due to its compressibility.

• Relief Zone: Includes delicate areas such as the palatine raphe and incisive papilla, which must be preserved to avoid excessive compression, (Fig. 1).

These zones are essential not only for retention but also for ensuring comfort and functionality of the denture (9). Detailed understanding of ridge anatomy and control over support zones are fundamental for both conventional impressions and the transition to digital workflows.

The impression process in complete dentures is divided into two phases: preliminary (anatomical) impression and final (functional or corrective) impression (10,11). Preliminary impressions are taken using stock trays for edentulous arches with high-viscosity materials such as alginate or compound to capture the full extent of the arch and displace soft tissues. Although this step may compress and distort the fibromucosa, it is only used to fabricate the custom tray. Using a customized tray, the functional impression records the topography and resilience of the fibromucosa under functional conditions. Materials such as border molding compounds (for peripheral seal), zinc oxide-eugenol pastes, and flowable silicones are used to ensure precision and minimize distortion (10).

Impression techniques vary among compressive, mucostatic, and selective pressure methods. Compressive impressions are used to deform the mucosa in resilient areas like the hard palate but may cause pain and bone resorption. Mucostatic impressions aim to reduce tissue compression and are suitable for areas with thin, delicate fibromucosa. Relief areas are created on the model to avoid compressing the mucosa. Selective pressure impressions combine both approaches, compressing resilient areas (e.g., soft palate) while preserving delicate regions. This technique is particularly effective when using materials like zinc oxide-eugenol paste and condensation silicone, ensuring precision and minimizing distortions (10).

Advances in impression techniques and materials for edentulous arches aim to maximize denture adaptation to the mucosa, promoting comfort and functional efficiency. This adaptation enhances physical principles essential for retention, such as adhesion, cohesion, surface tension, and atmospheric pressure, preventing denture displacement. Furthermore, proper peripheral sealing prevents air entry, improving retention (12). Support is achieved through extensive basal area coverage, precise adaptation, and uniform distribution of masticatory forces on resistant osseous areas, preventing overload and bone resorption. Stability depends on factors such as tooth positioning in the neutral zone, the relationship between the denture base and peri-prosthetic musculature, stable occlusion without premature contacts, and patient functional training (12).

Occlusal schemes significantly impact stability and patient satisfaction. While systematic reviews indicate that bilateral balanced occlusion and canine guidance have similar clinical efficacy, lingualized occlusion tends to yield higher satisfaction, whereas monoplane occlusion results are less favorable (13-16). Further robust studies are needed to confirm these findings.

History and Evolution of the Digital Workflow in Complete Denture Fabrication

The fabrication of complete dentures using the digital workflow has significantly developed since the first report by Maeda *et al*. (1994), when the use of CAD/CAM technology in prosthetics was still in its early stages (17). Initially, the accuracy of intraoral scans of edentulous arches was questioned, as scanners were unable to properly capture the areas of movable and resilient mucosa, as well as the smooth and shiny areas of the ridges (6,7,18). The lack of well-defined anatomical landmarks, such as the teeth present in dentate ridges, made it difficult to accurately read the ridges and overlap scans (18). These issues were gradually resolved with the evolution of scanners and software, especially after 2018, when technological advances allowed intraoral scanning of edentulous ridges to become significantly more accurate (19).

Recent studies have shown that the accuracy of intraoral scans of edentulous ridges is comparable to conventional impressions, with many highlighting the effectiveness of modern scanners. However, the techniques for scanning edentulous ridges still require care and precision. One of the most common approaches described in the literature begins with the impression of the ridge, which can be done either with intraoral scanning of the edentulous ridge or by scanning the conventional impression or the gypsum model (18,20,21).

Intraoral scanning must be done carefully to ensure that the adjacent soft tissues are properly retracted, allowing accurate reading of the basal area. The upper arch scan begins at the tuberosity on one side and follows the entire alveolar ridge to the tuberosity on the opposite side, returning to the midline at the anterior region, toward the incisive papilla. In the lower arch, the process is similar, starting at the retromolar papilla and following the crest of the alveolar ridge to the opposite side, including the vestibular and lingual slopes of the ridge (6,20-22).

Next, a trial base over the model is virtually designed, printed, and added to a wax plan. The orientation of the wax planes in the mouth and the determination and recording of the Vertical Dimension of Occlusion (VDO) and Centric Relation (CR) are done in a manner similar to the analog workflow. Outside the mouth, the articulated wax planes are scanned, with the entire inner surface of the trial bases and the vestibular and lingual surfaces of the wax planes being scanned, enabling overlay with the intraoral scan’s “Standard Tessellation Language” (STL) file (7,19,21).

The base of the denture and the teeth are designed using CAD software, employing teeth from digital libraries. The denture prototype is printed to test the aesthetics, phonetics, and comfort, with adjustments made as necessary before the final fabrication of the prosthesis. The prototype is typically printed in clear or white resins to facilitate aesthetic evaluation by the patient (7,19).

The final prosthesis can be milled or printed. Milling occurs in 5-axis milling machines using PMMA blocks for CAD/CAM. Typically, the base and teeth are milled separately and then bonded with rapid polymerization acrylic resin, but there are also bi-colored PMMA blocks that allow the fabrication of monolithic prostheses (22,23). Printed prostheses are made with liquid resins specific to 3D printers, with layers built up on a support and polymerized with ultraviolet or visible light. After completion, they undergo additional polymerization (curing) (24). They can also be fabricated by printing the base and teeth separately and then bonding them. The prosthesis is polished and glazed in a manner similar to the analog workflow and is ready to be installed (7,19).

Other approaches reported by some authors include the fabrication of individual trays via CAD/CAM. This technique uses a conventional preliminary impression, which can be scanned, either the impression or the gypsum model, or directly through intraoral scanning. The main advantage of this approach is the elimination of the ridge scanning step, as the individual tray is used to perform a conventional functional impression, which is later scanned (25). Bidra *et al*. (2016) and Mai, Lee (2020) report that by using a patient’s old prosthesis or a provisional prosthesis as the individual tray, the need to scan the ridge is eliminated (18,23). As a result, the number of clinical sessions can be reduced. The technique proposed by Mai, Lee (2020) allows the entire process to be completed in just two sessions, provided the patient is using prostheses that meet aesthetic criteria with the correct Vertical Dimension of Occlusion (VDO) and Centric Relation (CR), eliminating the try-in step (18).

Kouveliotis *et al*. (2020) used a stock denture trial base, relined with silicone to provide greater retention and stability. This relined base was used for the orientation of the wax planes and the maxillomandibular registration. The scan of the relined articulated bases was then overlaid with the initial intraoral scan, completing two clinical steps in one session (7).

In contrast, Srinivasan *et al*. (2021) followed a more traditional workflow, starting with the preliminary impression, followed by the functional impression, orientation of the wax planes, facebow registration, mounting of the models in an articulator, and teeth arrangement up to the try-in stage. After the wax denture setup was approved, the assembled set was scanned, and a replica of the wax prosthesis was milled or printed, completing the laboratory phase (26).

Recent Innovations and Technological Advancements in Complete Denture Fabrication

Recently, digital dentistry has benefited from significant innovations with the introduction of technologies that enhance precision and customization in the fabrication of complete dentures. One such innovation involves the use of virtual articulators, such as the Artex CR Virtual Articulator by Amann Girrbach (27). The use of the virtual articulator allows for precise mounting of the teeth without the need for a physical articulator. This approach facilitates the simulation of mandibular movements, contributing to a functional and accurate prosthesis, aligned with the digital design of the mounted teeth.

Another significant innovation described in the literature is the use of facial scanning as an auxiliary tool in denture fabrication. The technique proposed by Lo Russo *et al*. (2020,2021) involves scanning the middle and lower third of the patient’s face while they are smiling, using intraoral scanners (21,28). The goal is to capture the entire perioral area, including the tip of the nose, chin, and lower forehead. Additionally, they use a mobile application, the Bellus 3D FaceApp, to scan the entire face of the patient (28). These facial images can be overlaid onto the scans of the wax rims and edentulous ridges, creating a “digital facebow.” This integration of facial and dental information allows for greater customization and predictability of the prosthesis, improving the aesthetics and comfort of the patient. However, the overlay of facial and dental scans presents a technical challenge in edentulous patients, where the absence of clear anatomical landmarks makes the image alignment difficult (21,28). This technique, though complex, promises to revolutionize restorative dentistry, enabling dentures that are more aesthetically aligned with the patient’s natural smile.

Finally, the use of facial scanning technologies and advanced software represents a significant shift in the denture fabrication process, allowing for greater precision and customization, as well as a more detailed aesthetic planning that better meets the patients’ needs.

## Discussion

The digital workflow in Dentistry has become increasingly accessible, providing gains in efficiency, patient comfort, and cost reduction. It reduces clinical and laboratory time, the use of materials for impressions, and shipments between the office and the laboratory (29-31). However, the investment cost and learning curve remain barriers (30). Even without an intraoral scanner, it is possible to work with digital technologies through scanning services offered by some laboratories, which digitize physical models.

The concept of digital dentures was introduced in 1994 (17), although the intraoral scanning of edentulous ridges was only validated in 2018 (19). The fabrication of complete dentures faces challenges, especially in replicating the edentulous arch. Initially, intraoral scanning of edentulous areas was hindered by the mobility and texture variation of the mucosa (6,7,18,11). The accuracy of intraoral scanning compared to conventional impressions has been studied. The accuracy of intraoral scanning varies depending on the region of the arch, being more precise in areas of resistant mucosa and lower in dynamic areas, such as peripheral sealing (11,20,32,33).

Another relevant factor is that the adaptation and retention of digital complete dentures are still debated. Some studies suggest that the adaptation of digital dentures is similar to or superior to conventional ones due to mucostatic impressions (6,26,33-35). However, the retention of digital dentures still presents contradictory results. Some researchers point out that the retention of digital dentures is comparable to or superior to that of analog ones (26,34,36,37), while others indicate that conventional dentures offer better retention (25,32).

The discrepancy in results can be explained by variations in the CAD/CAM systems used, scanning techniques, and individual patient characteristics. Areas of resistant and keratinized mucosa tend to have better scanning accuracy (11), which favors adaptation. However, the retention of dentures depends not only on adaptation but also on peripheral sealing and atmospheric pressure (32,38).

The comparison between different types of materials used in the fabrication of denture bases is also relevant. Studies indicate that milled PMMA bases exhibit mechanical strength and dimensional stability similar to or superior to thermopolymerizable acrylic bases, in addition to avoiding polymerization shrinkage (26,33-35,39,40). Regarding 3D printing, although it is a more accessible technique, printed resin has shown inferior mechanical performance and durability (24).

As for patient acceptance, digital complete dentures have shown good feedback, with satisfaction equal to or superior to that of conventional dentures (6,25,28,34,41). Regarding phonetics, digital complete dentures are similar to or inferior to conventional ones (6,36). The masticatory force and efficiency of digital complete dentures are similar to or superior to conventional dentures (6,38).

The significant reduction in clinical time, one of the advantages of the digital workflow, is widely recognized (7,26,34,36,37). However, the number of clinical sessions for adjustments does not vary significantly between conventional and digital dentures (34).

## Conclusions

Within the limitations of this review, it is possible to conclude that:

1. The accuracy of intraoral scanning of edentulous ridges appears to be clinically acceptable, but there are still challenges regarding precision in areas of resilient mucosa;

2. The retention of digital complete dentures shows mixed results, and further studies are needed to validate whether they offer superior or equivalent performance to conventional dentures;

3. The use of additional technologies, such as facial scanning and virtual articulators, promises to improve the aesthetics and functionality of digital complete dentures, but the integration of these features with the digital workflow is still in the process of refinement;

4. Patient acceptance of digital dentures is positive, but further investigation is needed to explore the long-term impact of these dentures on phonetics and masticatory efficiency.

## Data Availability

The datasets used and/or analyzed during the current study are available from the corresponding author.
